# An Exaggerated Rise in Pulmonary Artery Pressure in a High-Altitude Dweller during the Cold Season

**DOI:** 10.3390/ijerph18083984

**Published:** 2021-04-10

**Authors:** Akylbek Sydykov, Abdirashit Maripov, Nadira Kushubakova, Kubatbek Muratali Uulu, Samatbek Satybaldyev, Cholpon Kulchoroeva, Djuro Kosanovic, Akpay Sarybaev

**Affiliations:** 1 Department of Internal Medicine, Excellence Cluster Cardio-Pulmonary Institute (CPI), Member of the German Center for Lung Research (DZL), Justus Liebig University of Giessen, 35392 Giessen, Germany; Akylbek.Sydykov@innere.med.uni-giessen.de; 2 Department of Mountain and Sleep Medicine and Pulmonary Hypertension, National Center of Cardiology and Internal Medicine, Bishkek 720040, Kyrgyzstan; ra.maripov@mail.ru (A.M.); nadira777@mail.ru (N.K.); kuba_med@mail.ru (K.M.U.); satybaldyev_91@mail.ru (S.S.); cholpon120393@gmail.com (C.K.); 3 Kyrgyz-Indian Mountain Biomedical Research Center, Bishkek 720040, Kyrgyzstan; 4 Department of Pulmonology, Sechenov First Moscow State Medical University (Sechenov University), 119992 Moscow, Russia; djurokos13@gmail.com

**Keywords:** high altitude, cold, pulmonary hypertension, echocardiography

## Abstract

Chronic hypoxia-induced sustained pulmonary vasoconstriction and vascular remodeling lead to mild-to-moderate elevation of pulmonary artery pressure in high-altitude residents. However, in some of them, severe pulmonary hypertension may develop. Besides hypoxia, high-altitude residents also face other environmental challenges such as low ambient temperatures. We describe a case of a 49-year-old woman of Kyrgyz ethnicity with abnormally increased pulmonary artery pressure, revealed by Doppler echocardiography. Significantly elevated pulmonary artery pressure was detected in late winter and this was not associated with right ventricular hypertrophy or right ventricular dysfunction. Repeat echocardiography performed in late summer disclosed a significant attenuation of pulmonary artery pressure elevation, with no changes in right ventricular performance parameters. This case illustrates that, in susceptible individuals, long-term cold exposure could induce an abnormal pulmonary artery pressure rise, which can be reversed during warm seasons as in our patient. In certain circumstances, however, additional factors could contribute to a sustained pulmonary artery pressure increase and the development of persistent pulmonary hypertension, which often leads to right heart failure and premature death.

## 1. Introduction

Excessive hypoxic pulmonary vasoconstriction is generally thought to contribute to marked pulmonary hypertension in sea-level residents with high-altitude pulmonary edema, and in high-altitude residents with chronic mountain sickness or high-altitude pulmonary hypertension [1,2]. Moreover, in chronic mountain sickness patients with slightly elevated resting pulmonary artery pressures compared to apparently healthy high-altitude dwellers, even light-intensity exercise was associated with an accentuated pulmonary artery pressure increase [3,4]. In sea-level residents, individual susceptibility to high-altitude pulmonary edema is associated with an abnormal pulmonary vascular response to hypoxia [5,6], and during exercise in normoxia [7,8]. Interestingly, subjects with a previous history of high-altitude pulmonary edema exhibited a more pronounced rise in pulmonary artery pressure and pulmonary vascular resistance in response to insertion of the hand and lower third of the forearm in crushed ice for two minutes compared to control individuals [9].

Thus, in addition to hypoxia and exercise, low ambient temperatures might contribute to an exaggerated pulmonary vascular response in predisposed individuals. Here, we report a case of a high-altitude dweller with cold-induced pulmonary hypertension.

## 2. Case Report

A 49-year-old woman of Kyrgyz ethnicity presented with exertional breathlessness during our late winter field expedition to a high-altitude area (3000 m, Sary-Mogol, Kyrgyzstan). She was a resident of a neighboring village, Taldy-Suu (3050 m). The patient had no medical conditions of note, no history of drug abuse or cigarette smoking and denied taking any medications. The physical examination showed a heart rate of 90 bpm, arterial blood pressure of 140 × 100 mmHg, and peripheral oxygen saturation (SpO_2_) of 88%. Respiratory, cardiovascular, and abdominal examinations did not reveal any pathological manifestations.

The complete blood count results were as follows: hemoglobin 16.6 g/dL, hematocrit 52%, red blood cell count 6.2 × 10^12^/L, platelet count 263 × 10^9^/L, and white blood cell count 8.1 × 10^9^/L. An electrocardiogram showed normal sinus rhythm, and no signs of right atrial or right ventricular hypertrophy. Pulmonary function tests assessed by spirometry were within the normal limits. Other methods of pulmonary function assessment such as lung volumes, diffusing capacity and imaging modalities were not available in such a remote high-altitude area.

**Figure 1:**
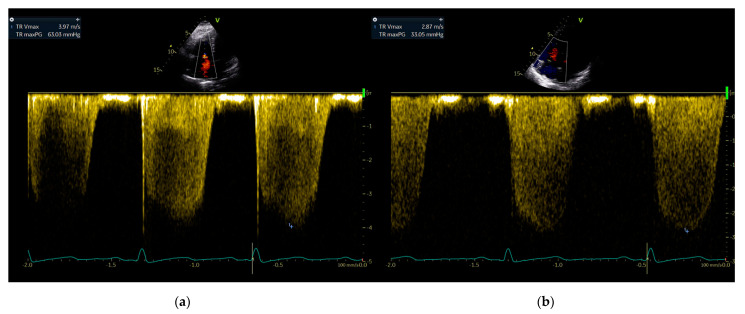
Echocardiogram in a patient with a high-altitude dweller, showing an exaggerated rise in pulmonary artery pressure during the cold season. Peak tricuspid regurgitation jet velocity estimating right ventricular systolic pressure measured in (**a**) the first examination in late winter, and (**b**) a repeat examination in late summer.

Doppler echocardiography revealed an increased tricuspid regurgitation peak gradient of 63 mmHg, indicating the presence of pulmonary hypertension (Figure 1a). The gold standard for the diagnosis of pulmonary hypertension is right heart catheterization. Unfortunately, this method is not available in remote high-altitude areas. In the last decades, significant technological advancements in echocardiography technology have increased its sensitivity for quantifying pulmonary artery pressure and it is now accepted as a safe and easily accessible alternative to right heart catheterization. Non-invasive echocardiography Doppler-based pulmonary artery pressure estimation has been demonstrated to be accurate vs. right heart catheterization [10]. In addition, the echocardiography examination was performed by an experienced sonographer, who considered potential sources of pulmonary artery pressure overestimation or underestimation in this patient [11].

No signs of right ventricular hypertrophy or dilatation were detected on the transthoracic echocardiogram. Right ventricular systolic function was not impaired, with the right ventricular fractional area change of 41%, tricuspid annular plane systolic excursion of 2.5 cm, peak systolic velocity at the lateral tricuspid annulus (S’) of 12 cm/s, and cardiac output of 5.8 L/min. All the investigations were performed in temperature-controlled rooms with the warm ambient temperature of 22−28 °C.

The patient was advised to move permanently to a lower-altitude location. However, she refused this recommendation due to family and economic reasons. The patient was invited for a follow-up investigation during our next field expedition to the high-altitude area in late summer. About six months later, she reported alleviation of the exertional breathlessness during summer time. Repeat physical examination did not reveal any major changes. The complete blood count results, electrocardiogram, and pulmonary function tests were not significantly different from the previous ones. A transthoracic echocardiogram showed a marked reduction in pulmonary artery pressure levels compared to the previous examination with a tricuspid regurgitation peak gradient of 33 mmHg (Figure 1b), while cardiac output and the parameters of the right ventricular function remained unchanged, they are as follows: right ventricular fractional area change of 44%, tricuspid annular plane systolic excursion of 2.4 cm, and S’ of 14 cm/s.

## 3. Discussion

Chronic hypoxia-induced sustained pulmonary vasoconstriction and pulmonary vascular remodeling lead to pulmonary artery pressure elevation in high-altitude residents, which is in most cases of mild-to-moderate degree [1]. However, in some of them, marked pulmonary hypertension may develop [12]. Besides hypoxia, high-altitude residents also face other environmental challenges such as low ambient temperatures [13,14]. In this regard, interesting observations were made on the association of the prevalence of severe hypoxic pulmonary hypertension and right heart failure development with cold weather in cattle after their exposure to high altitudes [15,16]. It was noted that the cattle were more frequently affected in winter, and during wet and cold summer; and the number of affected animals increased even more during hard winter [15]. Consistently, calves with an exaggerated hypoxic pulmonary vasoconstriction experienced more prominent effects of acute environmental cold on the pulmonary circulation [17]. On the other hand, sheltering animals from the cold attenuated pulmonary hypertension [17]. Nevertheless, exposure of non-susceptible cattle to the cold, in a temperature-controlled hypobaric chamber, also produced a significant increase in pulmonary arterial pressure and pulmonary vascular resistance at both moderate and high altitudes [18]. The effect of cold exposure on the pulmonary circulation was not potentiated by altitude [18]. Interestingly, cooling of the skin in cattle at thermoneutral ambient conditions increased pulmonary vascular resistance as well [19]. Collectively, these observations implied that both acute and chronic cold exposure might lead to pulmonary artery pressure elevation in cattle.

There is accumulating experimental evidence in favor of the detrimental effects of the cold on pulmonary circulation [20]. Acute hypothermia in anesthetized dogs has been shown to induce a progressive increase in pulmonary vascular resistance [21,22]. In awake rats, acute cold exposure in temperature-controlled chambers produced significant elevations in pulmonary arterial pressures [23]. These experiments also demonstrated that the magnitude of pulmonary artery pressure elevation was dependent on the degree of the temperature drop [23]. Long-term cold exposure in rats led to the development of persistent pulmonary hypertension [24]. Similar to other forms of pulmonary hypertension, chronic cold-induced pulmonary hypertension in rats was associated with increased medial layer thickness, narrowed lumen diameter of small pulmonary arteries, and right ventricular hypertrophy [25,26].

Importantly, a contributory or causative role of cold stress in the development of pulmonary hypertension or right heart hypertrophy has been shown for several other species, including guinea pigs [27,28], wild mice [29], sheep [30] and broilers [31,32,33]. In guinea pigs, chronic exposure to the cold was associated with the development of right ventricular hypertrophy, which can be considered as an indirect measure of pulmonary hypertension [27]. Furthermore, guinea pigs subjected to combined exposure to the cold and hypoxia exhibited significantly greater right ventricular weights than normoxic control animals [28].

Japanese scientists made interesting observations supporting the adverse effects of the cold on pulmonary circulation [29]. They investigated seasonal, latitudinal and global warming effects on the right ventricular hypertrophy indexes in wild wood mice [29]. The authors captured wild mice at the same place during different seasons and revealed seasonal changes in the degree of right ventricular hypertrophy [29,34]. In the summer, right ventricular hypertrophy indexes in these mice became smaller compared to those in winter, and they became greater again towards the winter. In addition, a strong negative correlation between environmental temperatures and right ventricular hypertrophy indexes was observed. There is an inverse relationship between latitude and temperature around the world, and the temperatures are typically cooler at higher latitudes. In order to evaluate the latitudinal effects, the authors compared right ventricular hypertrophy indexes in wild mice captured at two stations, Shirakabako and Hakkouda, in Japan. Shirakabako is located in the southern, warm region and Hakkouda is located in the northern, cold region. The mice inhabiting the warm regions had significantly lower right ventricular hypertrophy indexes than those inhabiting the cold regions [29]. In order to evaluate the global warming effects, the authors compared the data obtained in 1971 and in 2004. The authors noted that the average temperature increased over the past 30 years, probably due to global warming. In contrast, the right ventricular hypertrophy indexes in wild wood mice captured in 2004 were significantly lower compared to those captured in 1971 [29].

An earlier study showed that facial cooling produces an increase in pulmonary artery pressure and pulmonary vascular resistance in intensive care patients [35]. Interestingly, a recent echocardiography study conducted at sea level found that examinations performed in ambulatory patients during hotter months had a lower chance to show systolic pulmonary artery pressure >40 mmHg compared with those done in colder months [36]. In addition, an inverse correlation was demonstrated between several temperature indexes and systolic pulmonary artery pressure in these patients [36]. Interesting observations regarding the long-term effects of cold exposure on pulmonary circulation were made in the northeastern region of Russia [20]. Post-mortem investigations of the pulmonary vessels from immigrant workers and native residents found structural changes in the morphology of small pulmonary arteries, such as enhanced muscularization [37]. In addition to the observation of vascular remodeling of the pulmonary vessels, a high prevalence of pulmonary hypertension and right ventricular hypertrophy was revealed among the residents in this region [38].

Similar to these observations at sea level, healthy high-altitude residents responded to facial cooling with an increase in pulmonary vascular resistance [35]. We have recently shown that acute exposure to cold leads to the elevation of pulmonary artery pressure in high-altitude residents [39]. In line with these findings, highlanders exhibited slightly higher pulmonary artery pressure and pulmonary vascular resistance values at lower ambient temperatures than in a thermoneutral environment [40].

In our patient, the first echocardiographic examination performed in winter revealed significantly elevated tricuspid regurgitation peak gradient values. Repeat echocardiography conducted in late summer showed a significant alleviation of cold-induced pulmonary hypertension. These findings suggested that prolonged cold exposure in the winter probably induced structural remodeling in the pulmonary vessels. In the summer, warm ambient temperatures led to the reversal of pulmonary vascular remodeling, resulting in pulmonary hypertension amelioration. Remarkably, cold-induced pulmonary hypertension was not associated with right ventricular hypertrophy and dysfunction. We believe that the seasonal variations in pulmonary artery pressure levels might account for the lack of right ventricular hypertrophy and preserved right ventricular function in our patient.

Additional factors such as nutritional and socioeconomic conditions, seasonal migrations, geographical factors, which allow a significant change in altitude across a relatively short distance, have been suggested to modulate the impact of high altitude on erythrocytosis degree, pulmonary artery pressure levels and other physiological parameters in high-altitude dwellers [41,42,43]. Most Kyrgyz highlanders are pastoralists and practice seasonal transhumance. They move with their livestock between their permanent home, where they spend the harsh winter season, and summer pastures located at higher altitudes. Therefore, we speculate that, in a susceptible person, an initial increase in pulmonary artery pressure, which was probably induced by low ambient temperatures during the winter, would be maintained by more severe hypoxia at higher elevations during the summer. This would cause sustained pulmonary artery pressure elevation and the subsequent development of persistent pulmonary hypertension. On the contrary, because our patient does not practice transhumance, we revealed a significant pulmonary artery pressure decrease on repeat echocardiogram.

Thus, this case illustrates that, in susceptible individuals, long-term cold exposure could induce an abnormal pulmonary artery pressure rise. Although case studies are known for their limitations, such as, lack in ability to generalize, risk of over-interpretation, no possibility to establish cause–effect relationship, and retrospective design, case reports have always been a method of scientific communication in medical literature. Despite their limitations, case reports have the potential to contribute to research and change clinical practice through enhancing hypotheses generation and serving as the early phase of clinical research. Therefore, we are currently conducting a properly designed study to confirm the detrimental effects of long-term cold exposure on pulmonary circulation in high-altitude residents.

## 4. Conclusions

This case illustrates that, in susceptible individuals, long-term cold exposure could induce an abnormal pulmonary artery pressure elevation, which could be reversed during the warm seasons as in our patient. In certain circumstances, however, co-incidence of several predisposing or detrimental factors could lead to a sustained pulmonary artery pressure increase and the development of persistent pulmonary hypertension that often leads to right heart failure and premature death.

## Data Availability

The data presented in this study are available on request from the corresponding author.
